# Supplementary material for: The Antimicrobial Peptides Human β-Defensins Induce the Secretion of Angiogenin in Human Dermal Fibroblasts
Source: Int J Mol Sci. 2022 Aug 8;23(15):8800. doi: 10.3390/ijms23158800 (PMC9368840; doi:10.3390/ijms23158800)
Supplement: Supplementary file 1 [file ijms-23-08800-s001.zip › SupplementryData04.pdf]

Supplementary Data

The antimicrobial peptides human β-defensins induce the secretion of angiogenin in human dermal fibroblasts

Yoshie Umehara [1], Miho Takahashi [1,2], Hainan Yue [1], Juan Valentin Trujillo-Paez [1], Ge Peng [1], Hai Le Thanh Nguyen [1], Ko Okumura [1], Hideoki Ogawa [1] and François Niyonsaba [1,3,*]

[1] Atopy (Allergy) Research Center, Juntendo University Graduate School of Medicine, Tokyo 113-8421, Japan; y-umeha@juntendo.ac.jp (Y.U.); h-yue@juntendo.ac.jp (H.Y.); t-valentin@juntendo.ac.jp (J.V.T.-P.); g-peng@juntendo.ac.jp (G.P.); ha-nguyen@juntendo.ac.jp (H.L.T.N.); kokumura@juntendo.ac.jp (K.O.); ogawa@juntendo.ac.jp (H.O.); francois@juntendo.ac.jp (F.N.)

[2] Department of Dermatology and Allergology, Juntendo University Graduate School of Medicine, Tokyo 113-8421, Japan; m.takahashi@juntendo.ac.jp (M.T.)

[3] Faculty of International Liberal Arts, Juntendo University, Tokyo 113-8421, Japan

* Correspondence: francois@juntendo.ac.jp; Tel.: +81-3-5802-1591; Fax: +81-3-3813-5512

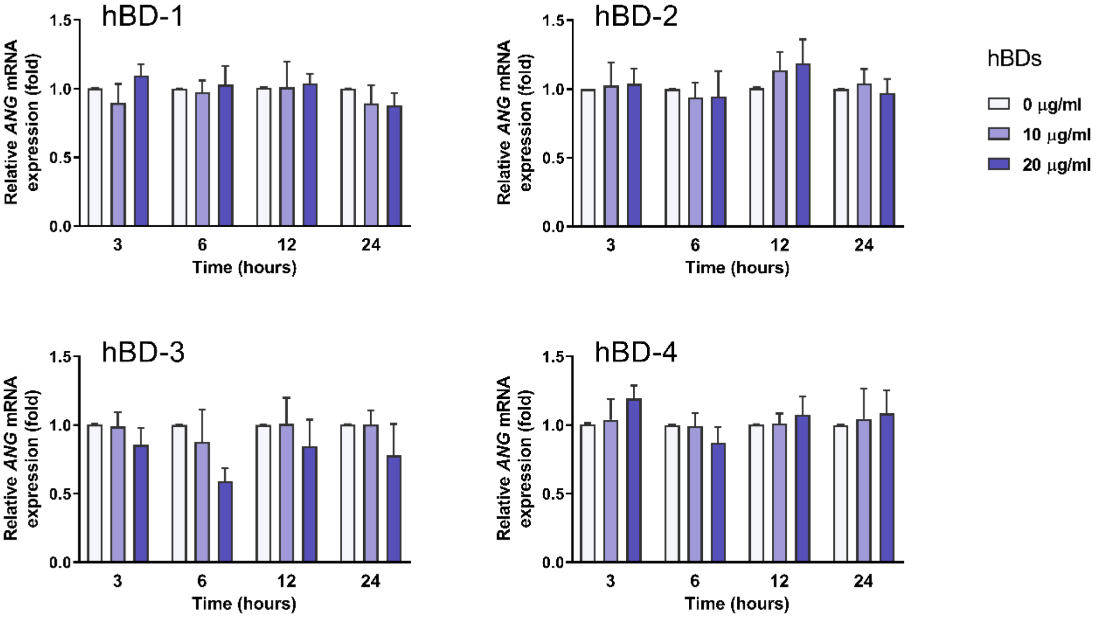

Supplementary Figure S1. The effect of hBDs on *ANG* expression by human dermal fibroblasts. Normal human dermal fibroblasts were stimulated with 10–20 μg/ml hBDs or vehicle (0 μg/ml hBDs) for 3–24 hours, and the expression of *ANG* mRNA was determined by quantitative real-time PCR. The data represent the means ± SDs of 4–6 separate experiments. There were no significant differences compared with the vehicle at each time point by one-way ANOVA with Tukey's multiple comparisons test.

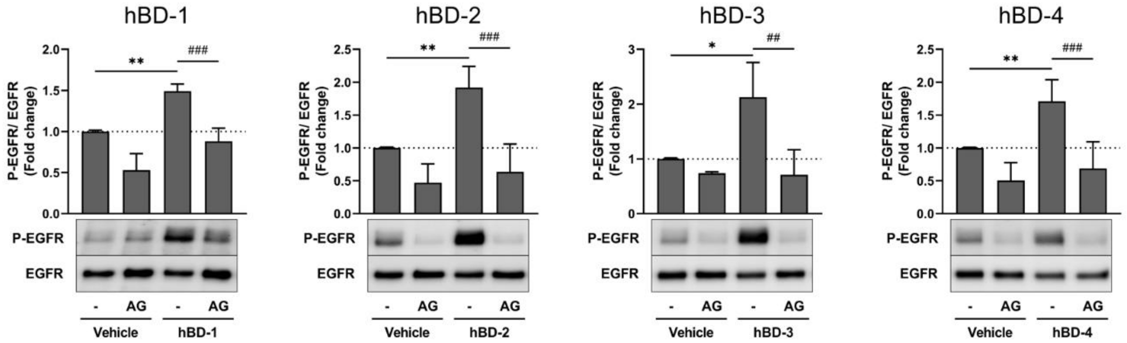

Supplementary Figure S2. EGFR inhibitor suppresses hBD-induced phosphorylation of EGFR. Normal human dermal fibroblasts were pretreated with 100 nM AG1478 (AG, EGFR inhibitor) or solvent (-) for 2 hours and then exposed to 20 μg/ml hBD-1, 20 μg/ml hBD-2, 10 μg/ml hBD-3 or 20 μg/ml hBD-4 for 60 minutes. The levels of phosphorylated and unphosphorylated EGFR in whole cell lysates were analyzed by Western blotting. *$P<0.05$ and **$P<0.01$ compared between the presence and absence of hBDs without inhibitors; ##$P<0.01$ and ###$P<0.001$ compared between the inhibitor-treated and untreated group with hBD stimulation by one-way ANOVA with Tukey's multiple comparisons test. All results are the means ± SDs of 4–6 independent experiments.

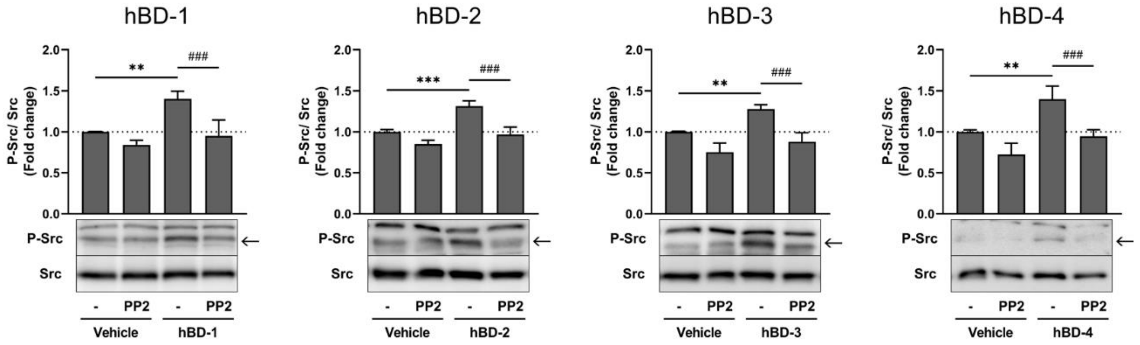

Supplementary Figure S3. Src inhibitor suppresses hBD-induced phosphorylation of Src. Normal human dermal fibroblasts were pretreated with 20 μM PP2 (Src inhibitor) or solvent (-) for 2 hours and then exposed to 20 μg/ml hBD-1, 20 μg/ml hBD-2, 10 μg/ml hBD-3 or 20 μg/ml hBD-4 for 60 minutes. The levels of phosphorylated and unphosphorylated Src in whole cell lysates were analyzed by Western blotting. **P<0.01 and ***P<0.001 compared between the presence and absence of hBDs without inhibitors; ###P<0.001 compared between the inhibitor-treated and untreated group with hBD stimulation by one-way ANOVA with Tukey's multiple comparisons test. All results are the means ± SDs of 4 independent experiments.

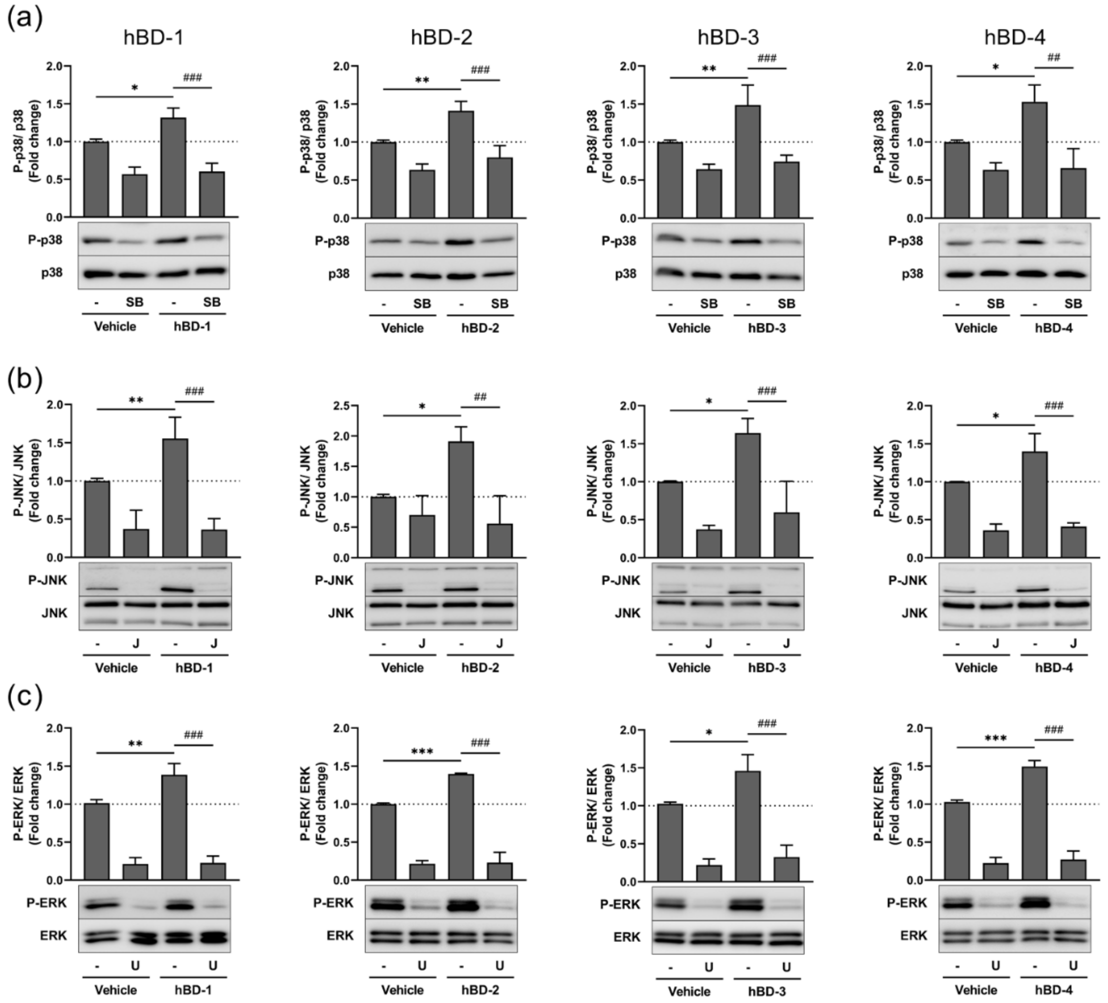

Supplementary Figure S4. MAPK inhibitor suppresses hBD-induced activation of MAPK signaling. Normal human dermal fibroblasts were pretreated with 10 μM SB203580 (SB, p38 inhibitor), 10 μM JNK in-hibitor II (J), 10 μM U0126 (U, ERK inhibitor) or solvent (-) for 2 hours and then exposed to 20 μg/ml hBD-1, 20 μg/ml hBD-2, 10 μg/ml hBD-3 or 20 μg/ml hBD-4 for 5 minutes. Whole cell lysates were analyzed by Western blotting to determine the levels of phosphorylated and unphosphorylated p38 (a), JNK (b) and ERK (c). *P<0.05, **P<0.01 and ***P<0.001 compared between the presence and absence of hBDs without inhibitors; ##P<0.01 and ###P<0.001 compared between the inhibitor-treated and untreated group with hBD stimulation by one-way ANOVA with Tukey's multiple comparisons test. All results are the means ± SDs of 3–4 independent experiments.

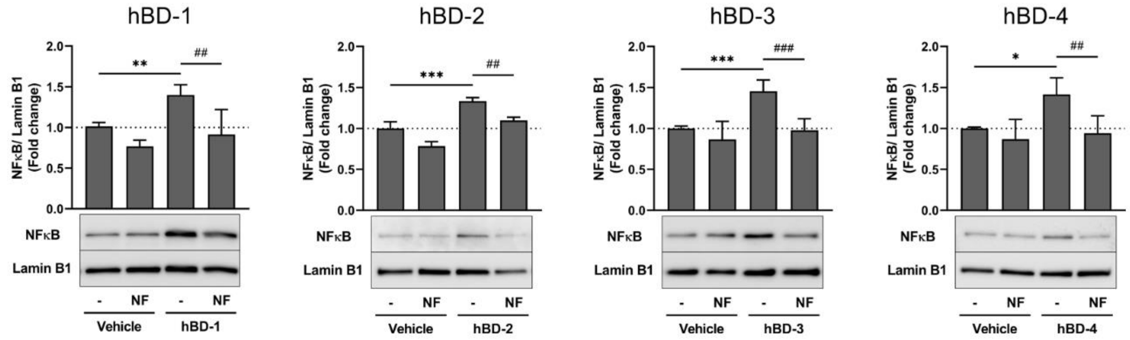

Supplementary Figure S5. NF-κB signaling inhibitor suppresses hBD-induced nuclear translocation of NF-κB. Normal human dermal fibroblasts were pretreated with 40 μM NF-κB activation inhibitor II (NF) or solvent (-) for 2 hours and then exposed to 20 μg/ml hBD-1, 20 μg/ml hBD-2, 10 μg/ml hBD-3 or 20 μg/ml hBD-4 for 60 minutes. The levels of NF-κB in nuclear lysates were analyzed by Western blotting. The expression of Lamin B1 is shown as a loading control. *P<0.05, **P<0.01 and ***P<0.001 compared between the presence and absence of hBDs without inhibitors; ##P<0.01 and ###P<0.001 compared between the inhibitor-treated and untreated group with hBD stimulation by one-way ANOVA with Tukey's multiple comparisons test. All results are the means ± SDs of 4–5 independent experiments.
