# Supplementary figures and images for: The Antimicrobial Peptides Human β-Defensins Induce the Secretion of Angiogenin in Human Dermal Fibroblasts
Source: Int J Mol Sci. 2022 Aug 8;23(15):8800. doi: 10.3390/ijms23158800 (PMC9368840; doi:10.3390/ijms23158800)

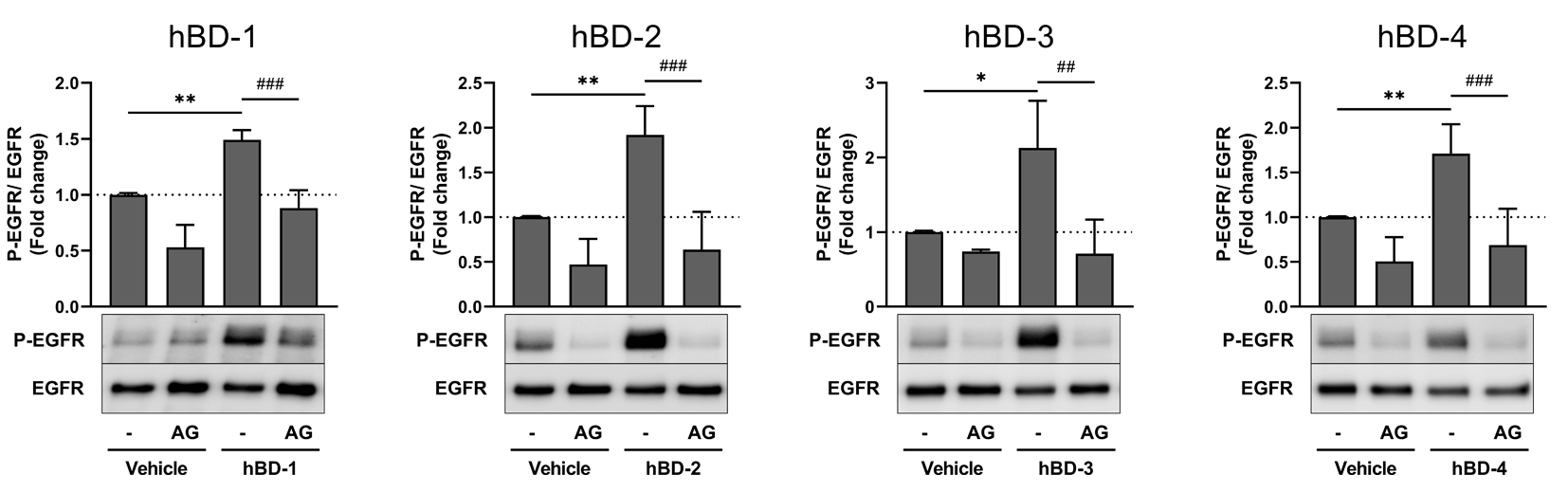

Supplement: Supplementary file 1 [file ijms-23-08800-s001.zip › FigureS2.tif]

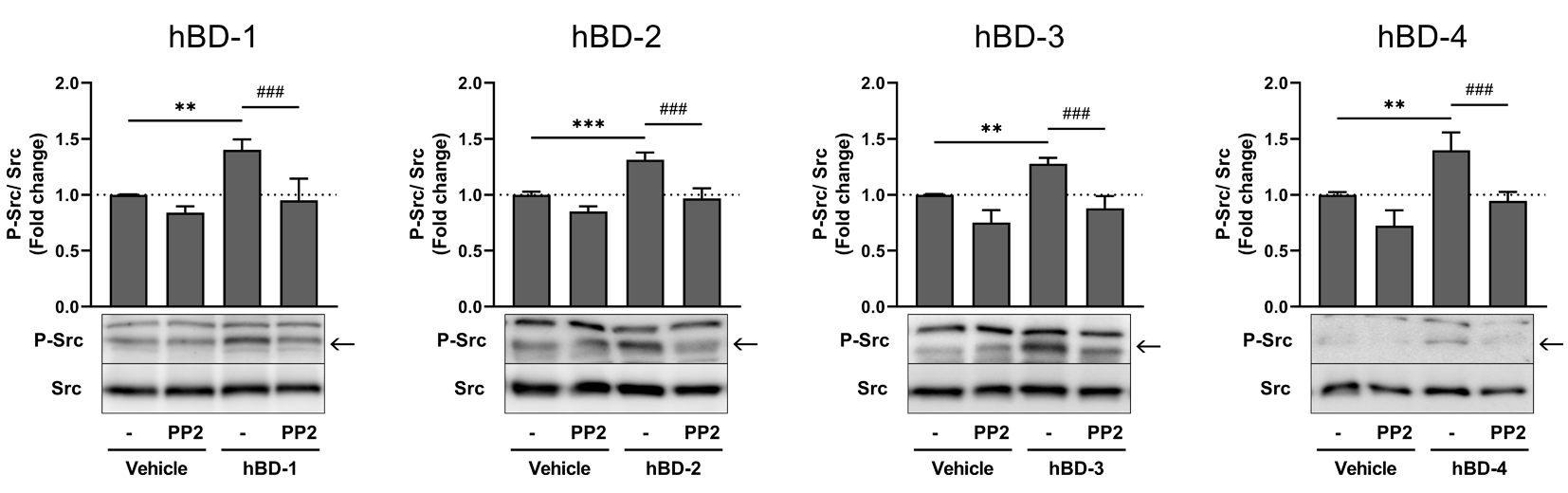

Supplement: Supplementary file 1 [file ijms-23-08800-s001.zip › FigureS3.tif]

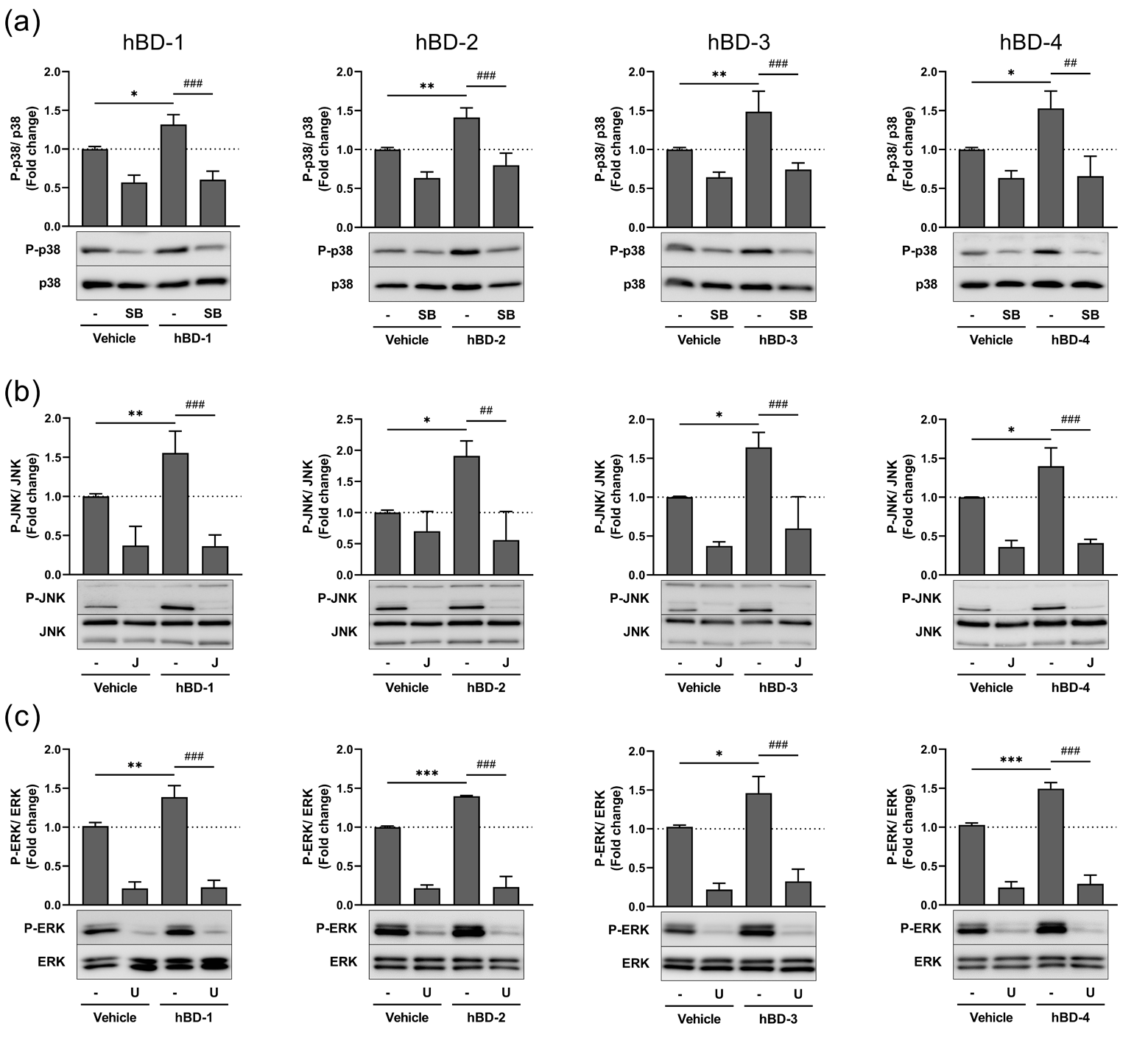

Supplement: Supplementary file 1 [file ijms-23-08800-s001.zip › FigureS4.tif]

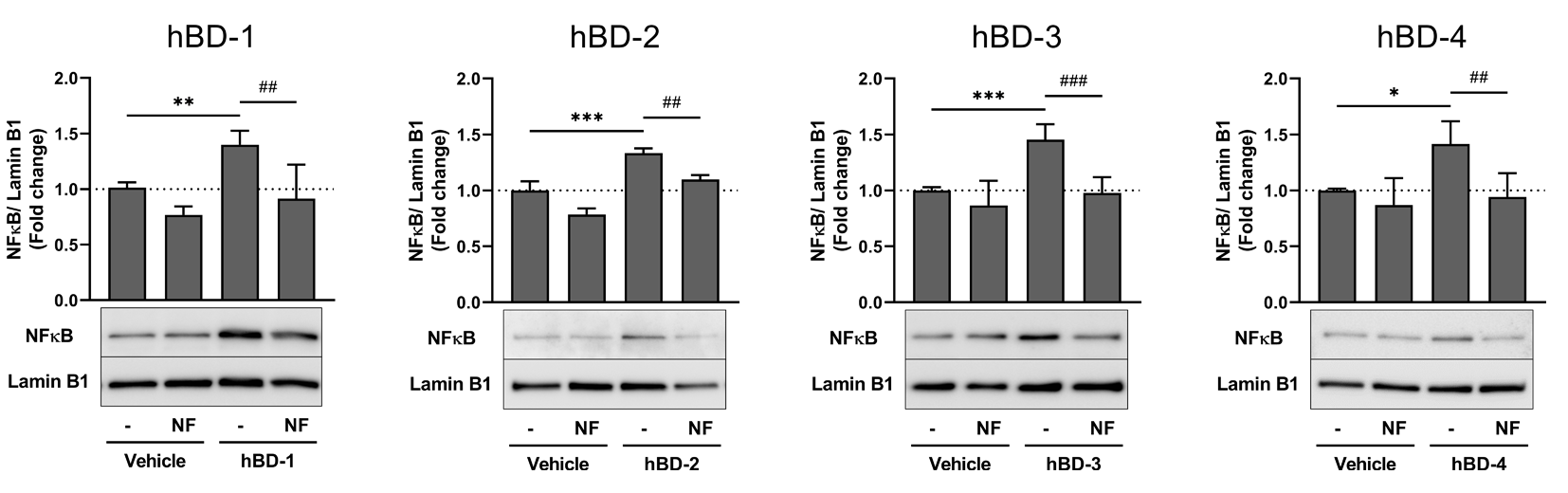

Supplement: Supplementary file 1 [file ijms-23-08800-s001.zip › FigureS5.tif]
